# Intralabyrinthine schwannomas: Surgical management and hearing rehabilitation with cochlear implants

**DOI:** 10.1007/s00106-017-0364-6

**Published:** 2017-06-29

**Authors:** S. K. Plontke, T. Rahne, M. Pfister, G. Götze, C. Heider, N. Pazaitis, C. Strauss, P. Caye-Thomasen, S. Kösling

**Affiliations:** 1 Department of Otorhinolaryngology, Head & Neck Surgery, Martin Luther University Halle-Wittenberg, University Medicine Halle, Ernst-Grube-Str. 40, 06120 Halle (Saale), Germany; 2 ENT Sarnen, Sarnen, Switzerland; 3 Institute of Pathology, Martin Luther University Halle-Wittenberg, University Medicine Halle, Halle (Saale), Germany; 4 Department of Neurosurgery, Martin Luther University Halle-Wittenberg, University Medicine Halle, Halle (Saale), Germany; 5 Department of Oto-rhino-laryngology, Head and Neck Surgery, and Audiology, University Hospital Rigshospitalet, Copenhagen, Denmark; 6 Department of Radiology, Martin Luther University Halle-Wittenberg, University Medicine Halle, Halle (Saale), Germany

**Keywords:** Acoustic neuroma, Hearing loss, Cochlear implant, Cochlea, Vertigo

## Abstract

Intralabyrinthine schwannomas (ILS) are a rare differential diagnosis of sudden hearing loss and vertigo. In an own case series of 12 patients, 6 tumors showed an intracochlear, 3 an intravestibular, 1 a transmodiolar including the cerebellopontine angle (CPA), 1a transotic including the CPA, and 1 a multilocular location. The tumors were removed surgically in 9 patients, whereas 3 patients decided for a “wait-and-test-and-scan” strategy. Of the surgical patients, 3 underwent labyrinthectomy and cochlear implant (CI) surgery in a single-stage procedure; 1 patient had extended cochleostomy with CI surgery; 3 underwent partial or subtotal cochleoectomy, with partial cochlear reconstruction and CI surgery (*n* = 1) or implantation of electrode dummies for possible later CI after repeated MRI follow-up (*n* = 2); and in 2 patients, the tumors of the internal auditory canal and cerebellopontine angle exhibiting transmodiolar or transmacular growth were removed by combined translabyrinthine–transotic resection. For the intracochlear tumors, vestibular function could mostly be preserved after surgery. In all cases with CI surgery, hearing rehabilitation was successful, although speech discrimination was limited for the case with subtotal cochleoectomy. Surgical removal of intracochlear schwannomas via partial or subtotal cochleoectomy is, in principle, possible with preservation of vestibular function. In the authors’ opinion, radiotherapy of ILS is only indicated in isolated cases. Cochlear implantation during or after tumor resection (i. e., as synchronous or staged surgeries) is an option for hearing rehabilitation in cartain cases and represents a therapeutic approach in contrast to a “wait-and-test-and-scan” strategy.

## Introduction

### Definition and classification

Intralabyrinthine schwannomas (ILS) are benign neoplasms originating from the peripheral branches of the cochlear nerve or the inferior or superior vestibular nerves. Because of their location and their management, they are a special subtype of vestibular schwannomas (acoustic neuromas) that typically occur in the internal auditory canal (IAC) and in the cerebellopontine angle (CPA). ILS are becoming particularly important in the differential diagnosis of cochleovestibular disorders and owing to their detectability via magnetic resonance imaging (MRI) examinations.

The tumors may be found in the cochlea, in the vestibule, or growing through the fundus into the IAC even up to the CPA. They may also grow from the inner ear into the middle ear and can present in different combinations of the aforementioned locations. There are several classifications, e. g., according to Jackler [11], who differentiates between “schwannoma of the vestibule” (also called “veritable vestibular schwannoma”), “schwannoma of the inner ear,” and “schwannoma of the inner ear and the internal auditory canal.” More detailed and more relevant classifications with respect to the management of these tumors were developed by Kennedy and co-authors [14] or by van Abel [31]. This article will refer to the classification suggested by Salzman and co-authors ([23]; Table 1).

**Table 1:** Classification of intralabyrinthine schwannomas according to location (modified from Salzmann et al. [23])

Classification	Description	Figure(s)
Intracochlear	Tumor limited to cochlea	1B, 1D, 3, 5, 6
Transmodiolar	Tumor in cochlea with extension to IAC via modiolus	1E
Intravestibular	Tumor in vestibule with or without extension to semicircular canals	1A, 2
Transmacular	Tumor in vestibule with or without extension to semicircular canals and extension to IAC macula cribrosa	–
Intravestibulocochlear	Tumor in vestibule with or without extension to semicircular canals and in the cochlea	–
Transotic ± CPA	Tumor in cochlea and/or in vestibular portion of the inner ear; extension into the middle ear and into the IAC with or without extension to CPA	1F, 8

**Fig. 1:**
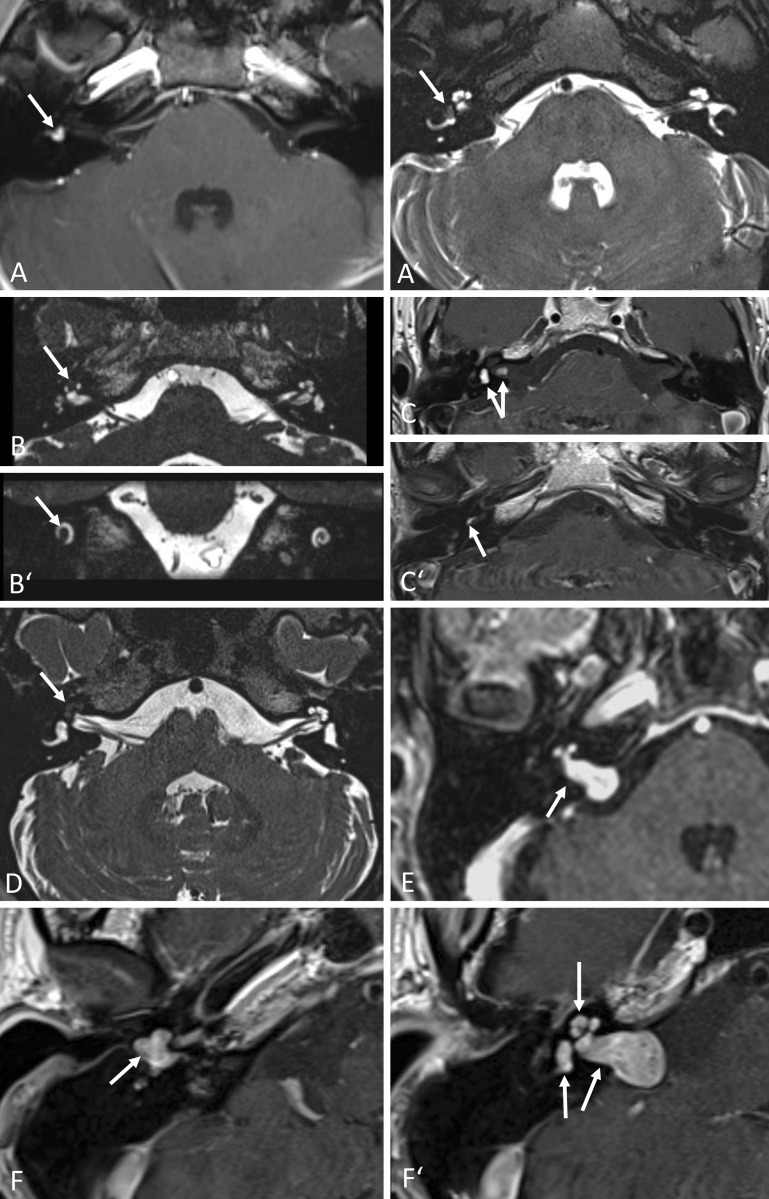
Intralabyrinthine schwannomas (ILS); MRI findings (*arrows*). **A,** **A′** Typical contrast enhancement on T1-w (**A**) and “filling defect” on T2-w (**A′**) images of histologically confirmed intravestibular schwannoma (patient 8). Axial images. **B,** **B′** Intracochlear schwannoma in the apical and partially in the middle turn (T2-w, **B** axial, **B′** coronal); not histologically confirmed (patient 4). **C,** **C′** Three locally separated schwannomas: intravestibular, intrameatal (**C**), basal cochlear turn (**C′**); not histologically confirmed (T1-w, c.m., axial; patient 6). **D** Intracochlear schwannoma of the entire cochlea, histologically confirmed (T2-w, axial; patient 9). **E** Tumor probably originating from the internal auditory canal (IAC) with extension into the cerebellopontine angle and transmodiolar into the basal and middle cochlear turn; histologically confirmed (T1-w, c.m., axial; patient 12). **F,** **F′** Transotic ILS with extension from the tympanic membrane via the entire labyrinth and the IAC to the cerebellopontine angle; histologically confirmed (T1-w, c.m., axial; patient 10). *w* Weighted, *c.m.* contrast medium

Not listed here are the tympanolabyrinthine (middle ear and entire inner ear) and translabyrinthine (entire inner ear and IAC), which are mentioned by van Abel et al. (2013). Multilocular tumors as in Fig. 1C are not mentioned in previous classifications

*IAC* internal auditory canal, *CPA* cerebellopontine angle

### Clinical symptoms

The clinical symptoms of patients suffering from ILS are not specific for the disease. Patients may present with symptoms similar to other cochleovestibular disorders. Nearly all patients with ILS suffer from hearing loss, which may occur acutely (often misdiagnosed as “idiopathic sudden sensorineural hearing loss”), develop slowly or progressively, but can also be fluctuating. Generally, hearing loss is sensorineural, however, conductive or combined hearing loss has also been reported. Balance problems (dizziness, vertigo, postural instability) present a mixed picture and symptoms may be similar to those of hydropic ear diseases [9]. Vestibular problems are more often observed in patients with ILS (especially in intravestibular findings) than in cases of schwannomas of the IAC and the CPA. Furthermore, tinnitus and a sense of pressure in the ear may occur [4, 5, 12, 18, 24, 28].

### Diagnostics

**Fig. 2:**
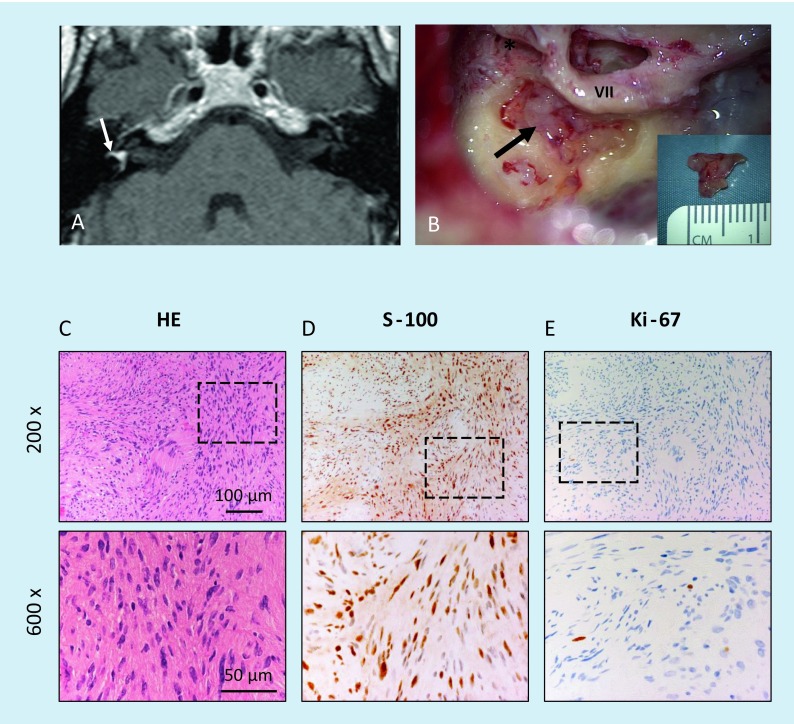
**A** Intravestibular schwannoma with growth in the vestibule and beginning into the semicircular canals (*arrow*; T1-w, c.m., patient 2). **B** Intraoperative view after opening of the labyrinth before tumor resection (*arrow*) and removal of the tumor (*inset*). **C** Histology and immunohistochemistry confirmed the intravestibular tumor as a schwannoma, showing compact spindle-shaped cells arranged in intersecting short bundles according to an Antoni-A pattern as well as a focal formation of nuclear palisades around nuclear-free areas (*upper row, center of the figure*); H&E. **D** Strong nuclear and weaker cytoplasmic positivity in immunostaining for S‑100. **E** Low proliferative activity of the tumor cells of max. 1% according to immunostaining for Ki-67. The *lower row* of figures shows magnifications of the areas marked in the *upper row*. *w* Weighted, *c.m.* contrast medium,* VII* facial nerve, mastoid course, *incus, *HE* hematoxylin & eosin

**Fig. 3:**
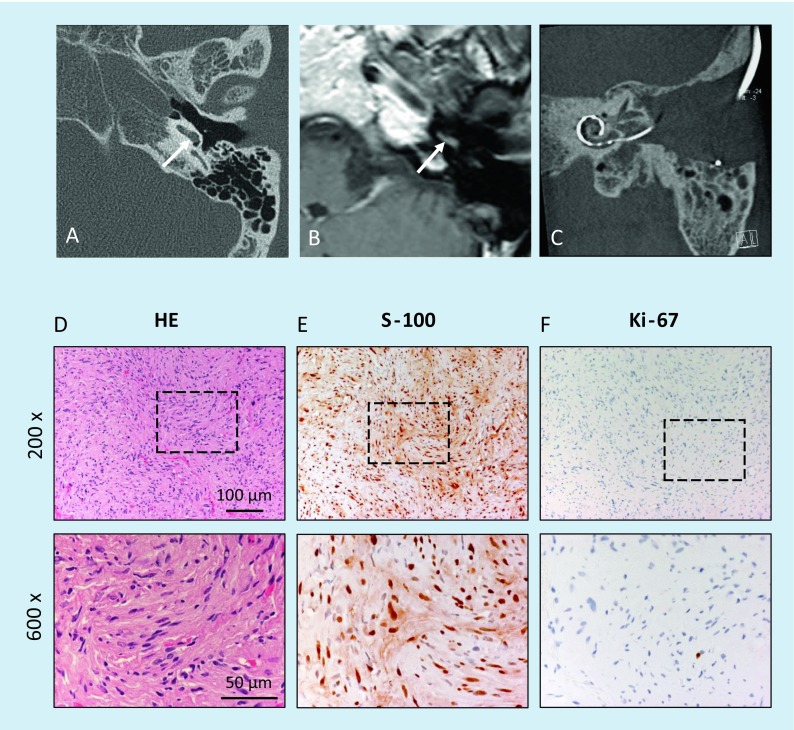
Intracochlear schwannoma in the basal cochlear turn (patient 3). **A** Normal findings on axial computed tomography (CT) scan of the temporal bone. **B** Tumor identification on MRI (T1-w c.m., axial). **C** Postoperative coronal cone beam CT with cochlear implant electrode carrier. Histological and immunohistological examination. **D** Histomorphological and immunohistochemical confirmation of the intracochlear schwannoma. The tumor tissue shows markedly cellular areas composed of elongated, spindle-shaped cells with fine fibrillary cytoplasm and with oval or cigar-shaped nuclei, corresponding to an Antoni-A growth pattern; H&E. **E** Strong nuclear and lower cytoplasmic positivity in the majority of the tumor cells in immunostaining for S‑100. **F** Low Ki-67 proliferation index of max. 1%. The *lower row* of figures shows magnifications of the regions marked in the *upper row. w* Weighted, *c.m.* contrast medium, *HE* hematoxylin & eosin

ENT-specific examination with ear microscopy and audiological diagnostic testing with pure-tone and speech audiometry, as well as neuro-otological diagnostic testing with functional testing of the semicircular canals and the otolith organs, should be performed on all patients with the aforementioned cochleovestibular symptoms. MRI represents the gold standard for diagnosis. The tumors typically show gadolinium contrast enhancement on T1-weighted images and a “filling defect” on T2-weighted images (Figs. 1, 2, 3, 5 and 6; [15, 23, 28, 29]). Based on our own case series, the present article describes experiences with this tumor entity, focusing on management and hearing rehabilitation with a cochlear implant (CI).

**Fig. 4:**
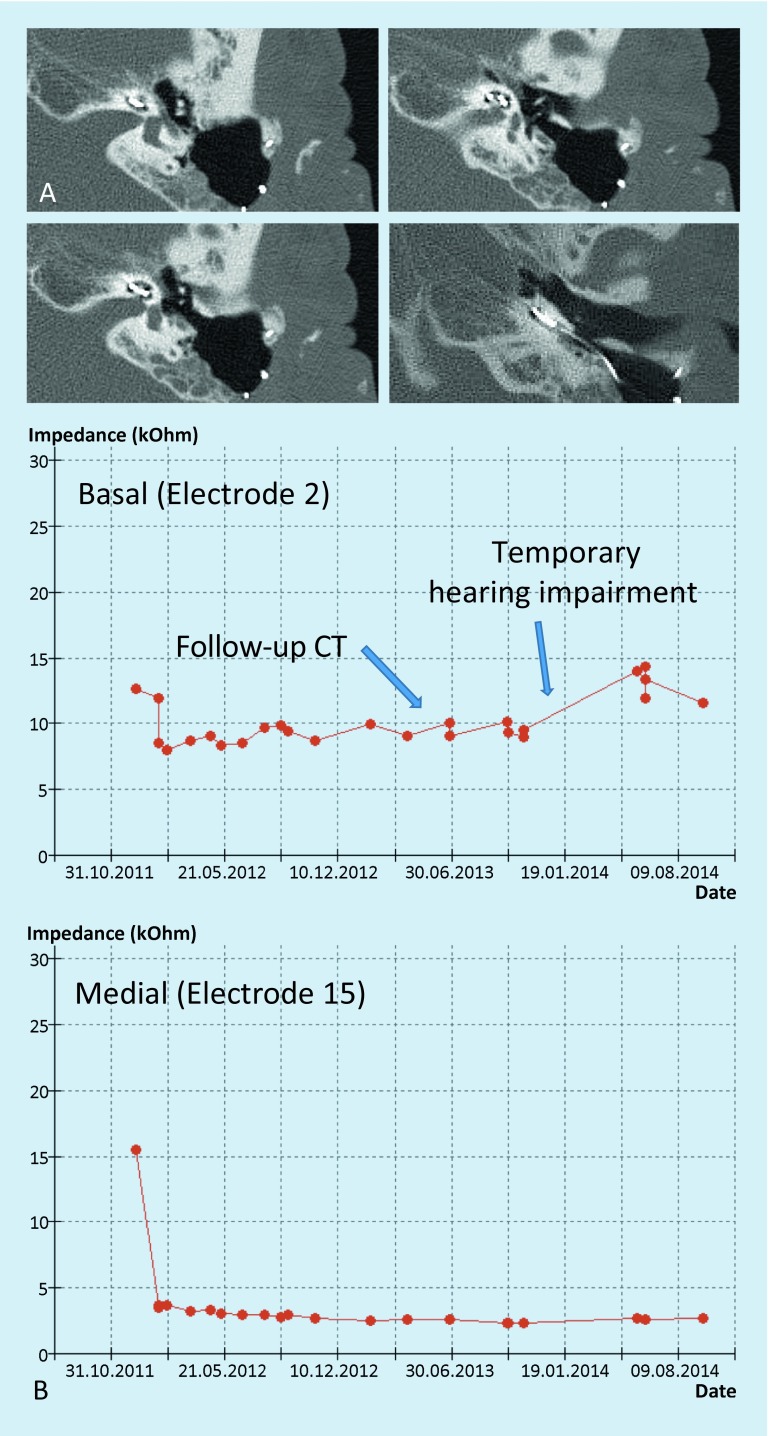
Postoperative follow-up of patient 3 (see Fig. 3). **A** Axial CT scans of the temporal bone without arrosion of bony structures. **B** Impedance measurement of the CI electrodes in addition to psycho-acoustic measurements

**Fig. 5:**
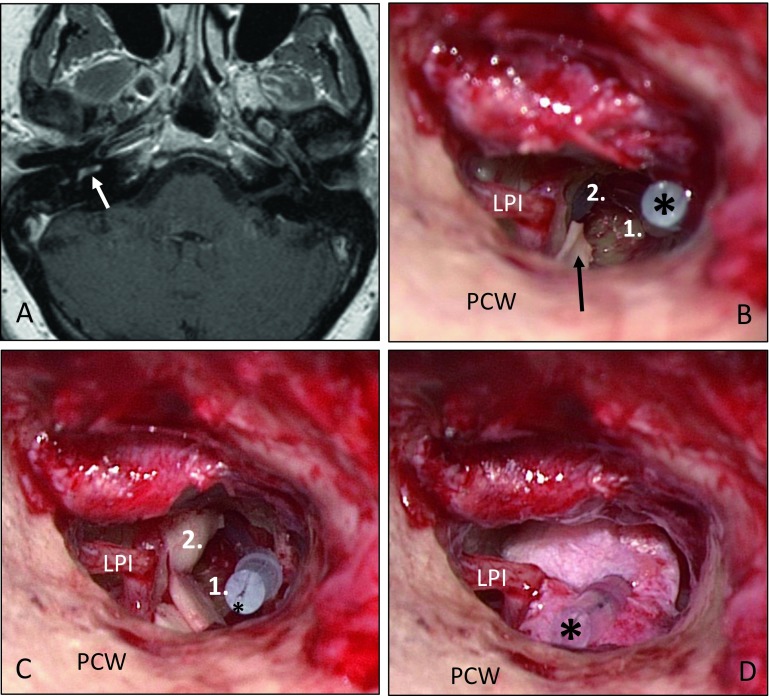
Partial reconstruction of the cochlea with cartilage and fascia after microscopically and mini-endoscopically [19] assisted transmeatal removal of an intracochlear schwannoma of the right basal turn (patient 7). **A** Histologically confirmed intracochlear schwannoma (T1-w, c.m., axial; *arrow*). **B** The placeholder (*“dummy electrode carrier”) is located in the basal (*1.*) and the second turn (*2.*). The vestibulum is covered with a small cartilage disc (*arrow*). **C** Reconstruction of the delimitation of the first and second turns is performed with cartilage (*2.*). **D** Finally, the reconstruction is covered with fascia. *LPI* Long process of incus, *PCW* posterior canal wall

**Fig. 6:**
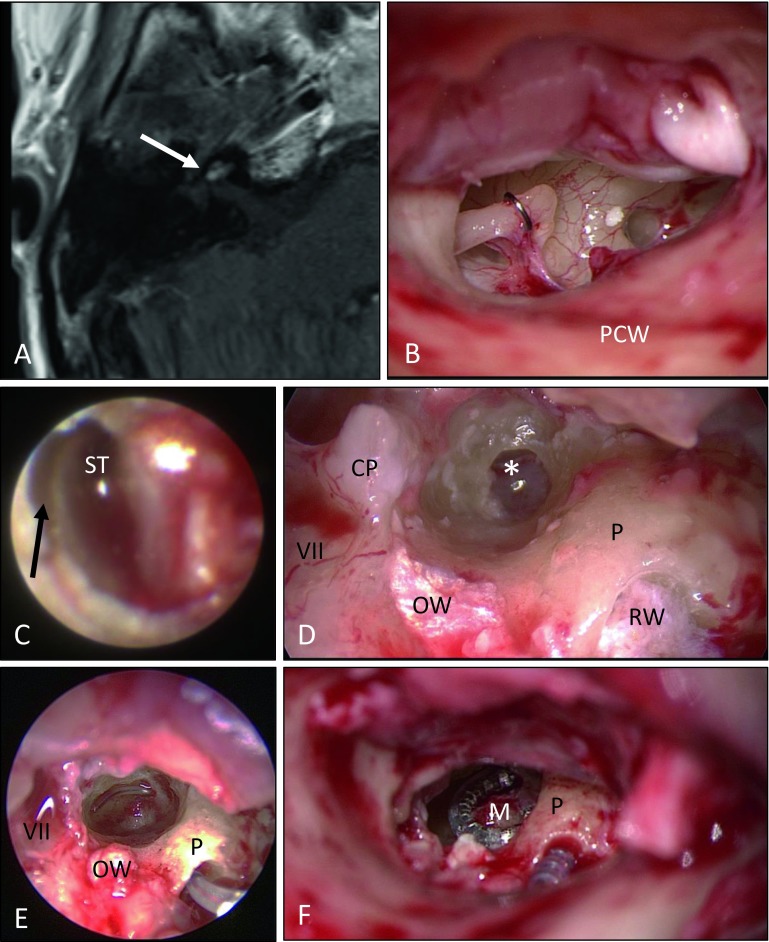
Intralabyrinthine schwannoma (ILS) in the right cochlea. Tumor resection via subtotal cochleoectomy and cochlear implantation (patient 11). **A** Tumor mass (*arrow*) in the middle and partially in the apical turn as well as partially in the basal turn (not shown) with contrast enhancement on MRI (T1-w, axial). **B** View of the middle ear with situation following implantation of a stapes wire prosthesis after Schuknecht about 15 years earlier. **C** View of the scala tympani (*ST*) by means of mini-endoscope [19] without any signs of a tumor in the basal part of the basal cochlear turn. The basilar membrane is seen as a dark line (*arrow*). **D** Opening of the cochlea in the anterior–inferior part of the cochleariform process reveals the tumor (*). The round window (*RW*) and the oval window (*OW*) are covered with fascia. **E** After subtotal cochleoectomy for tumor resection, only the lateral wall of the basal turn and a stump of the modiolus (*M*) are present. The temporarily inserted placeholder (“dummy electrode carrier”) is located at the cochlear wall (endoscopic view, 3mm, 0º). A CI with the electrode carrier placed close to the modiolus (Nucleus® CI 512 with Contour Advance electrode, Cochlear, Australia) was inserted (**F**) and the cochlea was partially reconstructed with perichondrium and bone pâté (not shown). *VII* Facial nerve; *PCW* posterior canal wall *P* promontory, *CP* cochleariform process

## Material and methods

The present series of 12 cases includes patients of our own case series (S.K.P.) with the first diagnosis of ILS made from 2008 to May 2016. Some patients were referred to us from other institutions. Except for patient 1, all patients underwent consultation and treatment (consultation and follow-up in cases of “wait-and-test-and-scan” strategy) at the Department of Otorhinolaryngology, Head and Neck Surgery of Martin Luther University Halle-Wittenberg. The clinical cases, symptoms, audiological, neuro-otological, radiological, and histological findings as well as the procedures are described in the context of a retrospective evaluation. Special focus is placed on surgical procedures including hearing rehabilitation with CI.

The demographic data, symptoms, and audiological and neuro-otological findings are summarized in Table 2, and tumor locations in Table 3.

**Table 2:** Demographic data and baseline parameters before therapy or when first presenting at our institution

Patient ID	m/f	Age^a^	Side	Initial symptoms	4PTA (dB HL)	WRS_65dB_ (%)	WRS_max_ (%)	4PTA contral. (dB HL)	Calorics DP (%)	vHIT gain (anterior; lateral; posterior)	cVEMP AR	oVEMP AR
1	m	38	R	Sudden hearing loss and vertigo	71	0	0	53.2	–	–	–	–
2	m	39	R	Provocational nystagmus, later low-frequency hearing loss and tinnitus	>110	0	0	21.25	–	–	0.92	0.81
3	m	49	L	1998: posttraumatic deafness contralateral, 10/2010: sudden hearing loss left followed by fluctuating hearing loss; HA left; tinnitus R > L, no vertigo/dizziness	108.75	0	0	>110	34.7	–	–	–
4	m	44	R	Tinnitus, progressive hearing loss, pressure sensation/fullness	75	0	25	16.25	0	–	–	–
5	m	43	L	Progressive hearing loss, tinnitus	54	30	80	5	–	–	–	–
6	f	50	R	Sudden hearing loss with deafness	>110	0	0	17.5	–	–, 0.70, –	–	–
7	f	60	R	Since 2011 progressive hearing loss (recurrent episodes of sudden hearing loss), 8/2013 deafness, episodic vertigo for approximately 24 h, tinnitus	>110	0	0	7.5	32.1	–	–	–
8	f	25	R	10/2014: sudden hearing loss; tinnitus; attacks of spinning vertigo, 3–4 x per day	55	5	80	8.75	32.8	0.46; 0.68; 0.65	0.35	0.69
9	f	37	R	2005: sudden hearing loss; postural instability, progressive hearing loss to deafness (2015)	>110	0	0	6.25	3.6	0.92; 0.99; 0.85	0.69	n.a.
10	m	51	R	Sudden deafness 21 years ago, MRI at that time described as normal, tinnitus R > L	>110	0	0	8.75	24.1	0.42; 0.55; 0.46	–	–
11	f	59	R	1994: stapedoplasty ipsilateral, 1994 MRI described as normal; progressive hearing loss, HA, history of breast cancer	111.25	0	15	10	only warm irrigation tolerated	0.43; 1.17; 0.66	–	–
12	f	65	R	Hearing loss bilateral since 1988, since 1992 HAs, since 2015 HAs insufficient	93.75	0	0	>110	7.7	not possible (ocular episthesison vHIT camera side)	0.32	0.34
Σ	6 f 6 m	47 ± 10	10 R 2 L	–	–	–	–	–	–	–	–	–

*m/f* Male/female, *AR* asymmetry ratio, *R* right, *L* left,* DP* directional preponderance, – no measurement done, *HA(s)* hearing aid(s), *4PTA* pure-tone average at 0.5, 1, 2, 4 kHz, *WRS*_*65dB/max*_ (word recognition score): percent of monosyllables (Freiburger test in quiet) understood at 65 dB sound pressure level (SPL) or maximally reached at an optimal stimulation level, *vHIT* video head impulse test, *VEMP* ocular (o) or cervical (c) vestibular evoked myogenic potentials, *deafness* refers to profound hearing loss or anacusis (no measurable threshold)

^a^Age in years at initial diagnosis by MRI (mean ± standard deviation)

**Table 3:** Interventions and results

Patient ID	Tumor location	Figure no.	Management	CI type	Postop. Calorics DP (%)	Postop. vHIT Gain (Anterior; Lateral; Posterior)	Postop. WRS_65dB_ (%)
1	Intravestibular	–	Tu-Ext. via labyrinthectomy + CI	Med-ElSonata	n.a.	n.a.	55
2	Intravestibular	2	Tu-Ext. via labyrinthectomy + CI	Med-El Mi1200 Flex28	n.a.	n.a.	45
3	Intracochlear (basal)	3, 4	Tu-Ext. via extended cochleostomy + CI	Cochlear CI24RE (CA)	–	0.85; 1.02; 0.91	90
4	Intracochlear (apico- und mediocochlear)	1B	W&T&S	–	n.a.	n.a.	n.a.
5	Intracochlear (apical)	–	W&T&S	–	n.a.	n.a.	n.a.
6	Multilocular: intravestibular, intrameatal, intracochlear (basal)	1C	W&T&S	–	n.a.	n.a.	n.a.
7	Intracochlear (basal)	5	Tu-Ext. via partial cochleoectomy, reconstruction and CI dummy insertion	Dummy	–	–	n.a.
8	Intravestibular	1A	Tu-Ext. via labyrinthectomy + CI	Med-El Mi1200 Flex28	n.a.	n.a.	25
9	Intracochlear	1D, 7	Tu-Ext. via subtotal cochleoectomy, reconstruction and CI dummy insertion	Dummy	36.3	0.77; 1.02; 1.17	n.a.
10	Transotic and CPA	1F,8	Tu-Ext. translabyrinthine/transotic, blind sack closure of EAC	Cochlear nerve and cochlea not preserved	n.a.	n.a.	n.a.
11	Intracochlear	6	Tu-Ext. via subtotal cochleoectomy, reconstruction and CI	Cochlear CI 512 (CA)	0	0.74; 1.11; 0.75	0
12	Transmodiolar and CPA	1E	Tu-Ext. translabyrinthine (transmastoidal) and transotic (transmeatal)	Cochlear nerve and cochlea not preserved	5.2	–	n.a.

*W&T&S* “wait-and-test-and-scan,” *WRS* 6–12-month postoperative word recognition score (monosyllables in quiet at 65 dB SPL), *vHIT* video head impulse test, *CI* cochlear implant, *DP* directional preponderance, *Tu-Ext.* tumor extirpation, *EAC* external auditory canal, *CPA* cerebellopontine angle, *n.a.* not applicable

**Fig. 7:**
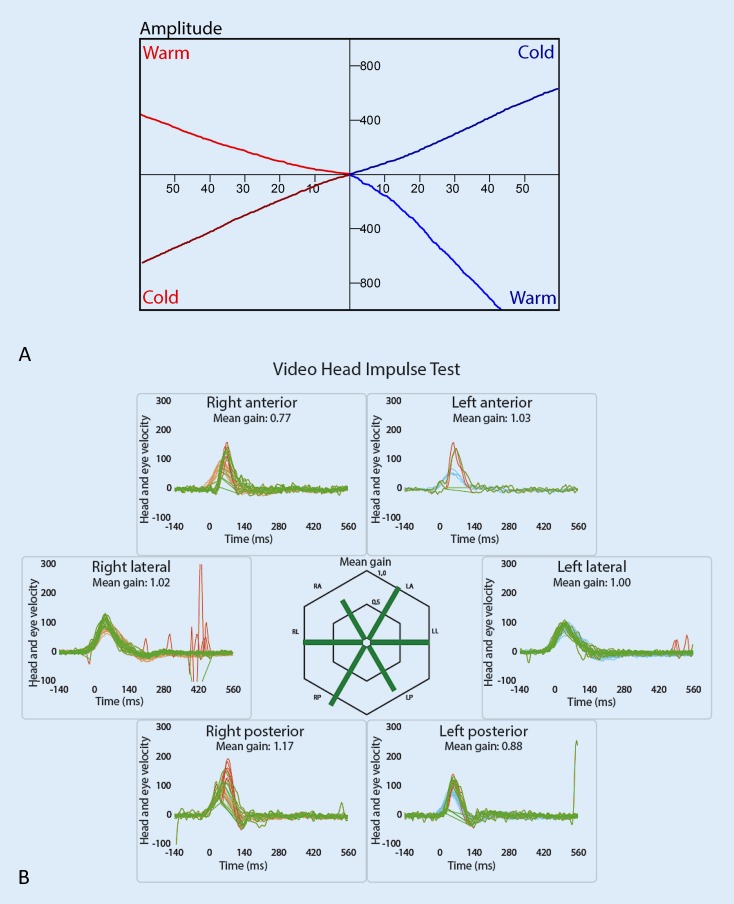
Preserved function of the semicircular canals 6 months after subtotal cochleoectomy, insertion of a dummy electrode carrier as well as partial cochlear reconstruction with cartilage and fascia (patient 9, Fig. 1D). **A** Caloric test, **B** video head impulse test

**Fig. 8:**
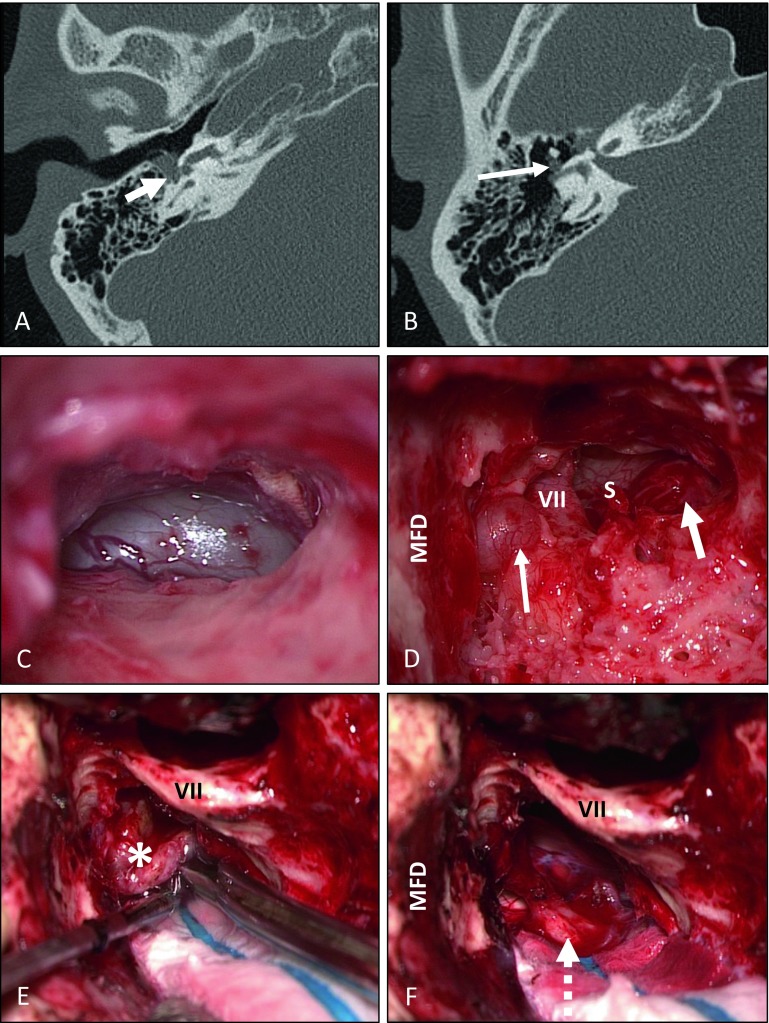
Translabyrinthine–transotic removal of a schwannoma extending from the cerebellopontine angle to the tympanic membrane. MRI studies performed about 23 years earlier because of contralateral tinnitus and 21 years earlier because of acute ipsilateral deafness did not show any signs of vestibular schwannoma/acoustic neuroma (based on written reports, images not available). The tumor that had likely developed as an ILS eroded the lateral semicircular canal with extension into the antrum (**B**, **D**, *thin arrow*) and the basal cochlear turn in the direction of the carotid artery (not displayed). It had also grown through the round window (*short arrow* in **A** and **D**) into the middle ear (**C**). (**A,** **B** axial CT scan of the temporal bone.) **C** Tympanoscopy for transmeatal biopsy.** E,** **F** Translabyrinthine resection of the tumor (*) in the internal auditory canal and the cerebellopontine angle with complete preservation of the facial nerve (*dotted arrow*). *MFD* dura of the middle fossa, *VII* facial nerve, *S* stapes

Audiological examinations were performed by means of pure-tone audiometry (4PTA as mean hearing threshold in the frequencies of 0.5, 1, 2, and 4 kHz) and speech audiometry (German Freiburger monosyllable test in quiet at 65 dB SPL level and maximal word recognition score).

For functional diagnostic evaluation of the otolith organs, vestibular evoked myogenic potentials (VEMP) were used. The difference in amplitude between sides with cervical (cVEMP) and ocular (oVEMP) recordings of the potentials was assessed.

Nine patients underwent surgery under general anesthesia. Two patients received implantation of a dummy electrode (insertion electrode, Med-El, Innsbruck, Austria) as off-label use. This procedure is possible and was discussed before surgery with the patients, who preferred it over single-step cochlear implantation [21]. Five patients underwent tumor removal together with cochlear implantation in a single-stage procedure.

## Results

The mean age of patients at the time of the first diagnosis made with MRI was 47 years (SD ± 11 years). Six patients (50%) were female and six (50%) were male. The present case series comprised six intracochlear tumors (50%), three intravestibular tumors (25%), one multilocular tumor (separate intravestibular and cochlear as well as intrameatal), one transmodiolar tumor with extension to the CPA, and one transotic tumor including involvement of the CPA (8% each). In 10 cases (83%) the tumor was located on the right and in two cases (17%) on the left side.

The management of the patients including the surgical procedures, the CI (if applicable), and the postoperative audiological and neuro-otological results are summarized in Table 3.

In three patients, labyrinthectomy was performed in combination with cochlear implantation (see Fig. 2 as an example). Another patient underwent tumor resection via posterior tympanostomy and extended cochleostomy as well as cochlear implantation as a single-stage procedure because of his strong wish with regard to professional rehabilitation (Fig. 3). Two patients had partial (patient 7) or subtotal (patient 9) cochleoectomy, partial reconstruction of the cochlea, as well as insertion of a dummy electrode carrier for repeated MRI follow-up in order to exclude tumor recurrence or the growth of a residual tumor (Fig. 5). Because of her own wish, one patient underwent tumor resection via subtotal cochleoectomy and partial reconstruction of the cochlea together with cochlear implantation in a single-stage surgery (Fig. 6). In two patients, translabyrinthine/transotic tumor resection was performed owing to tumor extension from the tympanic membrane or from the inner ear to the CPA (patients 10 and 12; Fig. 8).

**Fig. 9:**
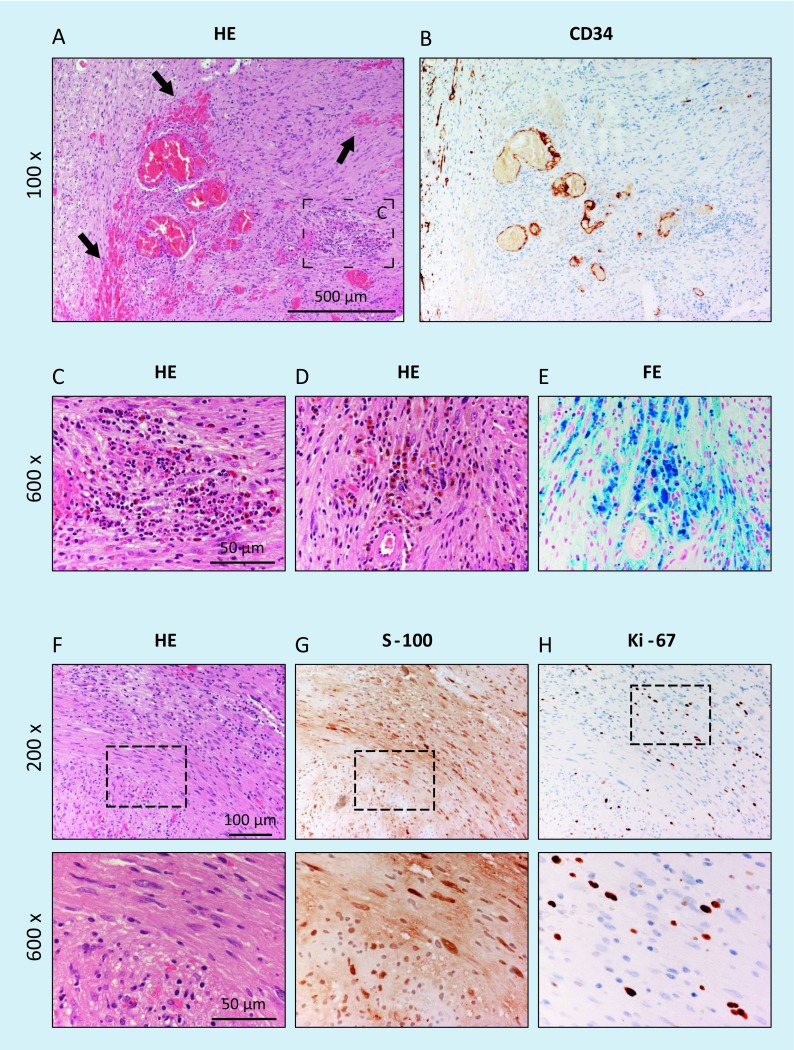
In most areas, the histological and immunohistochemical examinations of the tumor mass of patient 10 (Fig. 8) show the typical image of a schwannoma similar to the tumors of patients 2 and 3 (histology and immunohistochemistry described in Figs. 2 and 3). Moreover, degenerative changes such as residues of former bleedings in the form of interstitial hemosiderin deposits with hemosiderin-laden macrophages are seen, especially in tumor parts from the internal auditory meatus (**D,** **E**; identification of iron in the Prussian blue reaction; identical area on consecutive sections). Furthermore, fresh bleedings (*arrow* in **A**) as well as infiltration of acute inflammatory cells (neutrophil granulocytes in **C**) and prominent vascularization (CD34-positive endothelial cells in **B**) of the tumor are visible. **H** In the adjacent areas, there is a comparably high proliferative activity in the Ki-67 immunostaining focally of up to 10–12%. **F,** **G** However, histoarchitecture is present corresponding to a benign schwannoma with strong nuclear and weaker cytoplasmic expression of S‑100 (identical area on consecutive sections in **F** to **H**). *FE* Prussian blue reaction (iron detection)

The first symptoms of patient 10 were observed 23 years (tinnitus) and 21 years (deafness) before the diagnosis. Two MRI examinations performed at that time did not show any hint of vestibular schwannoma; however, when the patient presented to us, only the reports were available but no original images. In most tumor areas in this patient, histological and immunohistochemical examination showed typical findings of a schwannoma, characterized by spindle-shaped, elongated cells arranged in streams with diffuse expression of the marker protein S‑100 and a low proliferative activity (Fig. 9, comparable to the findings shown in Fig. 2C–E and Fig. 3D–F of patients 2 and 3). In other areas, however, characteristics were identified that may be considered as histomorphological correlates of a slowly progressive growth over many years. Here, degenerative tissue changes such as residues of former bleedings in the form of interstitial hemosiderin deposits with hemosiderin-laden macrophages (Fig. 9D/E) were seen beside fresh bleedings and focally also infiltration of acute inflammatory cells (Fig. 9A). Compared with the tumors of the other patients in this series, the tumor cells also revealed an increased proliferative activity in those areas (Fig. 9H) as well as more prominent vascularization (Fig. 9B). In summary, the histomorphological and immunohistochemical findings did not reveal malignant transformation of the schwannoma, as has also been described for patients without previous radiotherapy and without the presence of neurofibromatosis [26, 27].

After individual consultation and based on the patients’ wishes, three patients decided to pursue a “wait-and-test-and-scan” strategy.

A Schuknecht stapes prosthesis that was implanted about 15 years earlier had to be removed in one patient (patient 11) because otherwise no adequate approach to the intracochlear tumor would have been possible. One case of ILS with simultaneous otosclerosis had already been described in the literature [22]; however, it should be noted that ILS may also occur with combined hearing loss [28].

**Fig. 10:**
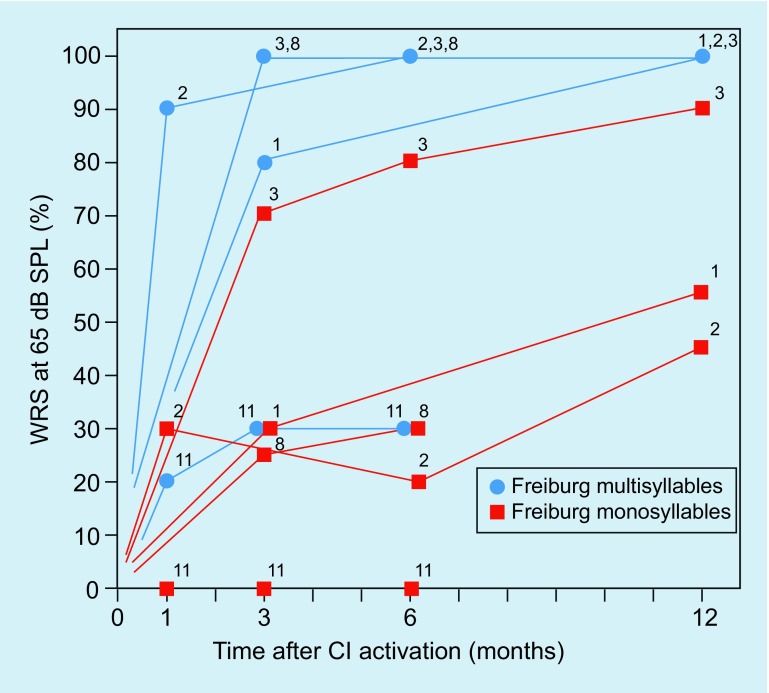
Audiological results of the patients with surgical resection of the ILS and cochlear implantation. The *numbers* in the graphs correspond to the number of the patients in Table 2. *SPL* sound pressure level, *WRS* word recognition score

After surgical removal of the intracochlear ILS (patients 3, 7, 9, 11), those patients with subtotal cochleoectomy (patients 9 and 11) suffered from temporary vertigo. The other two patients with intracochlear ILS (patients 3 and 7) did not complain of vertigo. Even after subtotal cochleoectomy, such as in patient 9, the preservation of the function of the semicircular canals is possible. Postoperative video head impulse test (vHIT) examination of patients 3 and 9 (Fig. 7), and patient 11 showed a regular gain in all three levels (Fig. 7). Otolith function testing by means of VEMPs was not possible in patients 9 and 11 because of conductive hearing loss due to the surgery-related missing incus (patient 9) or stapes and incus (patient 11). The audiological results of cochlear implantation are summarized in Fig. 10.

## Discussion

Despite the first descriptions of single cases in the literature over 40 years ago [10, 13, 32], the entity of ILS has been gaining increasing attention only in the last few years, likely due to continuous progress in the field of MRI technology and because of an increasing awareness and understanding of the importance of these tumors in the differential diagnosis of sudden hearing loss, chronic asymmetric hearing loss, unilateral peripheral vestibular disorders, or tinnitus. To date, because of the small size at the time of the first diagnosis, inappropriate MRI for diagnosis (e. g., head MRI instead of temporal bone MRI with too thick slices), and lack of awareness or not explicitly searching for an intralabyrinthine tumor on MRI, the incidence of these tumors is likely underestimated [4, 7, 31]. Thus, patients suffering from cochleovestibular symptoms should be evaluated for the presence of ILS by means of MRI. It seems reasonable to clearly mention the suspicion (or wish of exclusion) of an ILS when filling out the request form for radiologists or neuroradiologists.

Even if MRI initially does not (or does not definitively) reveal a vestibular schwannoma or an intralabyrinthine schwannoma, repeating the MRI (such as performed on patients 9 and 10 of this case series) seems to be feasible [21]. Because of missing data, no reliable recommendation can be made regarding the time of follow-up examinations. However, the authors consider an interval of 2–5 years as appropriate.

The “wait-and-test-and-scan” strategy is an option especially for patients without complaints [8, 18]. This includes patients without vertigo and those with sufficient auditory function (maximal understanding of monosyllables in a quiet environment ≥60%). In cases of an intracochlear location, however, one must bear in mind that tumor growth leads to increasing obstruction of the cochlea. This is a problem with regard to later rehabilitation with a CI. Tumor resection may then only be performed through a subtotal or total cochleoectomy (patients 9 and 11, Figs. 6 and 7). The cochlea is no longer available (or only to a very limited extent) for insertion of the CI electrode carrier. Furthermore, transmodiolar tumor growth into the internal auditory canal causes problems, since tumor resection with surgical preservation of the auditory nerve and spiral ganglia cells is no longer possible. An alternative to tumor resection or “wait-and-test-and-scan” is to leave the tumor in the cochlea despite insertion of the CI electrode carrier, as was described in three cases of primary ILS and in seven cases with neurofibromatosis and secondary intracochlear growth from the IAC. In such cases, even postoperative imaging by means of MRI could be applied for follow-up examinations [3]. If adopting a “wait-and-test-and-scan” strategy, the risks associated with the deposition of gadolinium-based contrast agents must be considered [6]. After initial diagnosis, however, it might not be necessary to apply contrast agent in each follow-up MRI. Intralabyrinthine growth may also be assessed by thin three-dimensional T2-weighted sequences.

Radiotherapy is also an option for the treatment of ILS. The indication, however, is considered to be very limited because of the good surgical accessibility of intralabyrinthine tumors. From the authors’ point of view, it is a preferred option for older patients with growing tumors. The single high-dose or fractionated radiation will generally inhibit the growth of “classic” vestibular schwannomas (internal auditory meatus and/or CPA). Sufficient experience has not been gained with small ILS. It must be suspected that radiotherapy of ILS damages the delicate sensory or neuronal structures, especially the cochlear spiral ganglia cells. This would lead to cochleovestibular functional impairment and an unfavorable prognosis regarding later CI rehabilitation. Additionally, there is a very low – but for younger patients relevant – risk of malignant transformation of the initially benign tumor [26, 27].

If symptoms like (functional) deafness and/or vertigo occur, surgical tumor resection is recommended [8, 18]. Microsurgery of intracochlear tumors usually leads to complete hearing [8]. In one case, however, even hearing improvement after surgical removal of an ILS was described in the literature [17].

The tumor location determines the procedure of microsurgical tumor resection. For tumors located in the vestibular part of the inner ear, labyrinthectomy is recommended with simultaneous cochlear implantation. Alternatively, an electrode dummy may be inserted, which clearly facilitates repeated MRI in order to exclude tumor recurrence or residual tumor growth. After labyrinthectomy in cases of translabyrinthine resection of intrameatal (with or without CPA) vestibular schwannoma, more than 50% of the patients experience early partial cochlear fibrosis [2]. Thus, we recommend inserting an electrode dummy as place holder or performing cochlear implantation in the same session in order to allow for hearing rehabilitation.

Tumors located in the cochlea may be resected via partial or subtotal/total cochleoectomy. In certain cases, partial “cochlear reconstruction” is possible usually after placement of a CI electrode carrier or a dummy electrode. However, only limited experience is reported to date. Possible material in this context might be cartilage to create “spaces” for insertion of an electrode carrier (in the sense of a scala or to delimit scalae from each other) and for sealing: perichondrium, fascia, and bone pâté (Fig. 5B and C; [21]). After implantation of a dummy electrode and repeated MRI, CI surgery may be performed at a later stage. Alternatively, a single-stage procedure is possible, i. e., cochlear implantation together with tumor resection [1, 3, 16, 20, 25]. Follow-up by means of MRI in such cases is more complicated or even impossible despite reports of successful imaging of the inner ear and the internal auditory canal for tumor control after cochlear implantation [3].

For tumors growing into the internal auditory canal and the CPA, resection is performed via a translabyrinthine approach. If the tumor does not extend to the cochlea and the hearing nerve is preserved, a CI electrode dummy should be inserted. After MRI follow-up, cochlear implantation may be performed after an interval. Alternatively, a single-stage procedure is possible; however, follow-up with MRI will be limited.

When specific factors such as implant position and MRI sequence are taken into account, the internal auditory canal and the inner can be visualized even after CI [30]. Furthermore, alternatives for follow-up should be checked, such as, e. g., electrophysiological and psycho-acoustic functional tests (electrode impedances, eABR, loudness scaling; Fig. 4).

Currently there are no – or only scarce – data available regarding tumor recurrence or growth rates of intraoperatively invisible residual tumor tissue after surgical resection of ILS. Furthermore, it must be taken into consideration that even independent from the removed ILS, new schwannomas – e. g., in the IAC or vice versa after removal from the IAC in the inner ear – may develop synchronously or metachronously as multilocular tumors (Fig. 1C). If cochlear implantation is performed in the interval, tumor recurrence or residual tumor, especially in the transmacular and transmodiolar space and in the fundus of the IAC, must be excluded by means of MRI prior to implantation. In cases where after cochlear implantation there is a clinical suspicion of tumor recurrence or the development of a secondary tumor, or if another disease develops that has to be examined by MRI, explantation of the CI might be required before MRI is performed.

## Conclusion

- ILS are a rare differential diagnosis of cochleovestibular disorders such as sudden hearing loss or hydropic ear disease.
- MRI examinations for sudden hearing loss should always include evaluation of the presence or absence of an ILS.
- Cochlear implantation during or after tumor resection (i. e., as synchronous or staged surgeries) is an option for hearing rehabilitation in ceratin cases and represents a therapeutic approach, in contrast to a “wait-and-test-and-scan” strategy.
